# Supplementary material for: Homologous basic helix–loop–helix transcription factors induce distinct deformations of torsionally-stressed DNA: a potential transcription regulation mechanism
Source: QRB Discov. 2022 Jun 10;3:e4. doi: 10.1017/qrd.2022.5 (PMC10392670; doi:10.1017/qrd.2022.5)
Supplement: Supplementary file 1 [file S2633289222000059sup001.zip › S2633289222000059sup001.pdf]

SUPPORTING INFORMATION

Homologous BHLH transcription factors induce distinct deformations of torsionally-stressed DNA: a potential transcription regulation mechanism

Johanna Hörberg, Kevin Moreau, and Anna Reymer*
Department of Chemistry and Molecular Biology, University of Gothenburg, Gothenburg 40530, Sweden
*E-mail: anna.reymer@gu.se.

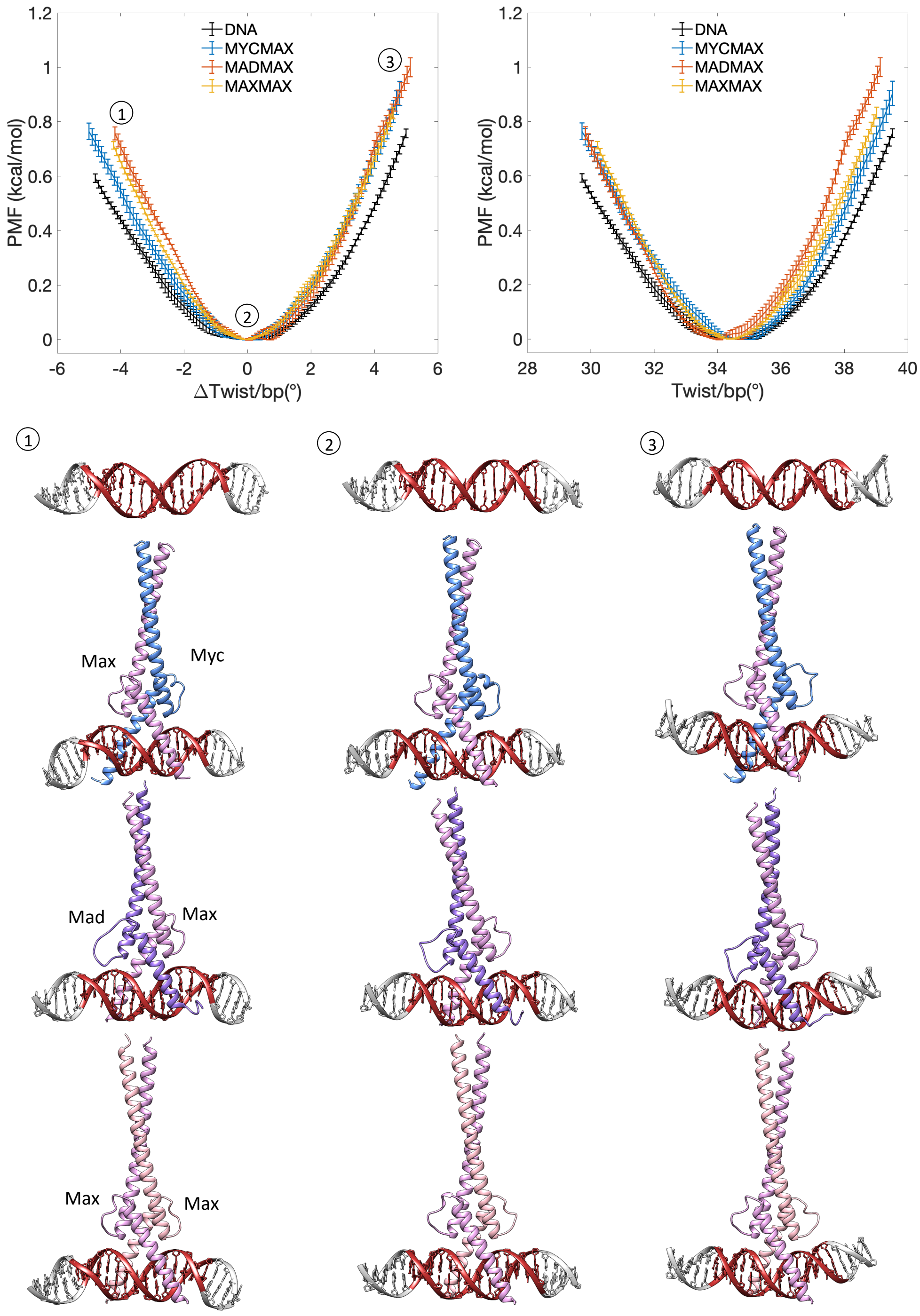

Figure S1: PMF profiles of DNA twisting transitions for unbound DNA and MycMax- (blue), MadMax- (orange) and MaxMax (yellow) bound DNA. Standard deviations of the 500 ns windows are highlighted as error bars. DNA deformations for unbound DNA, MycMax-, MadMax- and MaxMax-bound DNA are shown for (1) underwound regime (-4.5°/bp), (2) torsionally relaxed regime and (3) overwound regime(+4.5°/bp). The restrained DNA region (GTAGCACGTGCTAC) is highlighted with red colour.

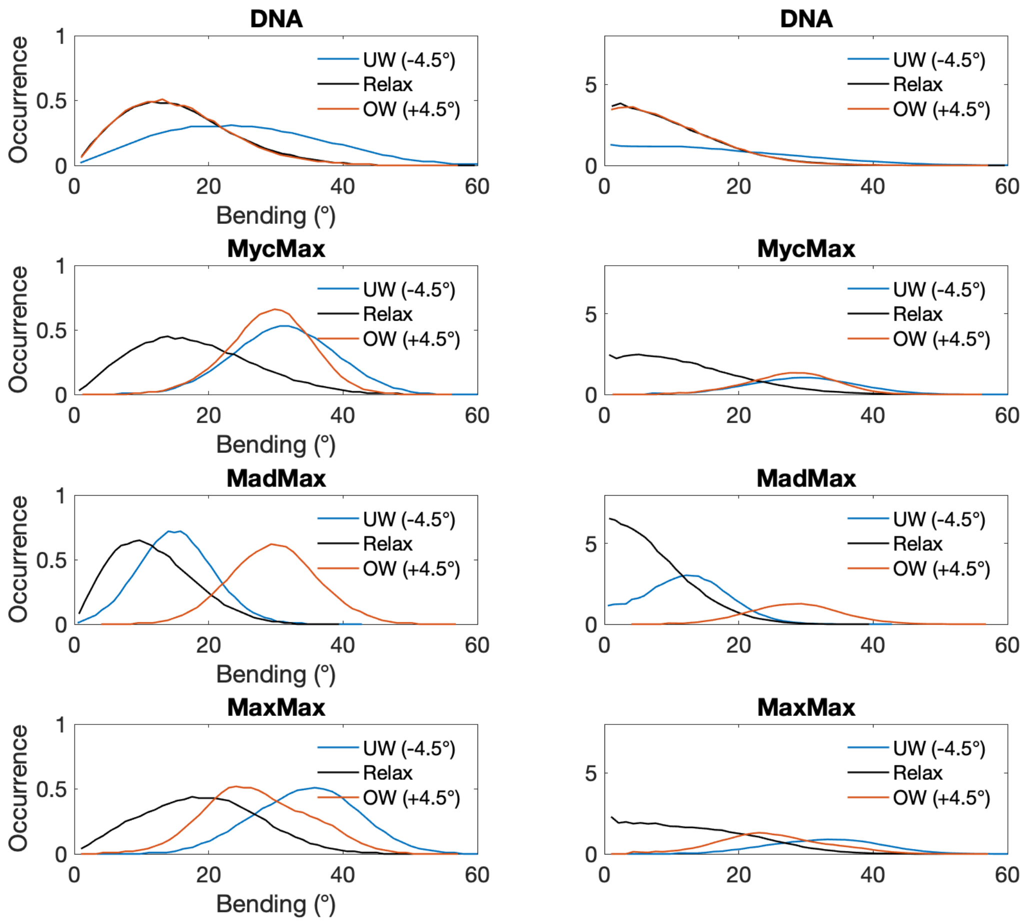

Figure S2: Bending distributions of the restrained region (GTAGCACGTGCTAC) for unbound DNA, MycMax-, MadMax- and MaxMax-bound DNA. For the right-hand panels, the histograms have been divided by sin($\boldsymbol{\theta}$), where $\boldsymbol{\theta}$ corresponds to the bending angle at the middle of each histogram bin, to compensate for the increasing area of the spherical ring segment sampled as the bending angle increases.

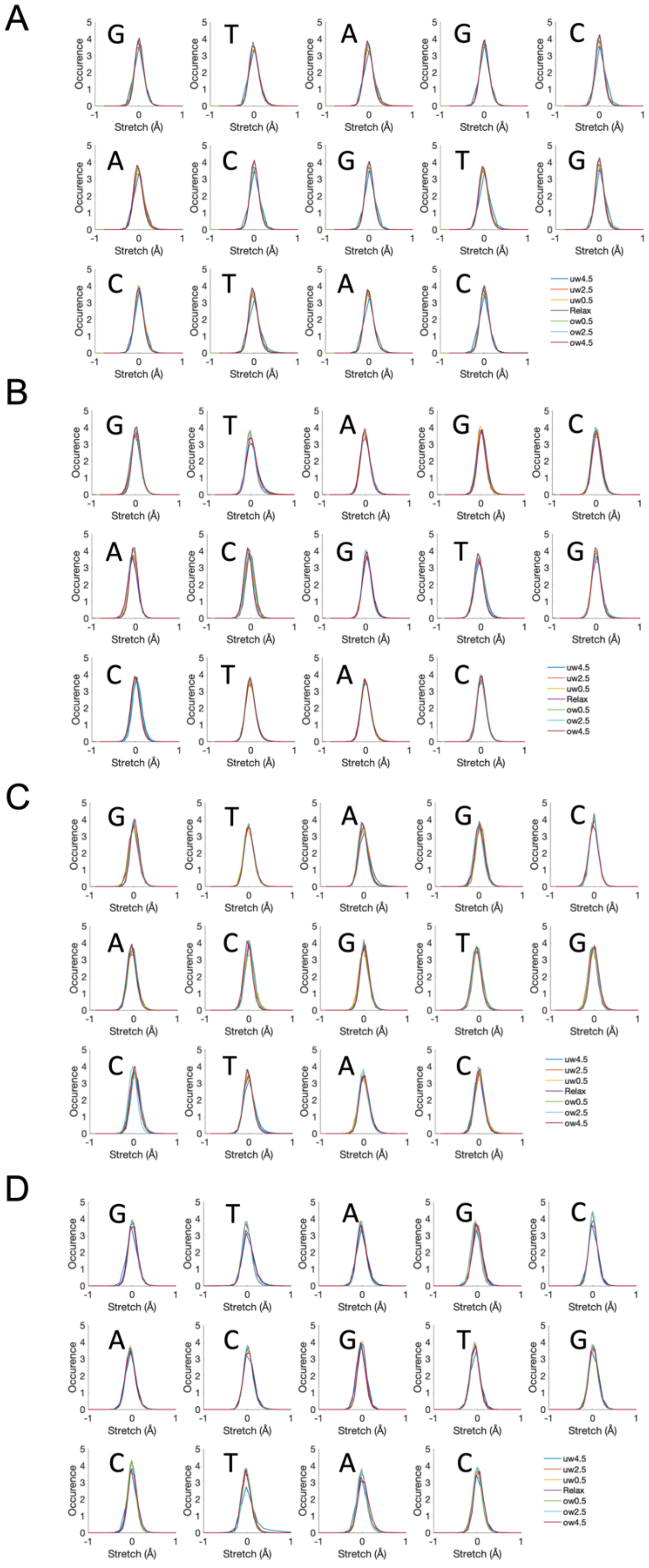

5'-GGCGA-**GTAGCACGTGCTAC**-TCGC-3'

Figure S3: Stretch distributions for b.p. of the restrained region for different torsional regimes. **A.** DNA, **B.** MycMax-DNA, **C.** MadMax-DNA, **D.** MaxMax-DNA.

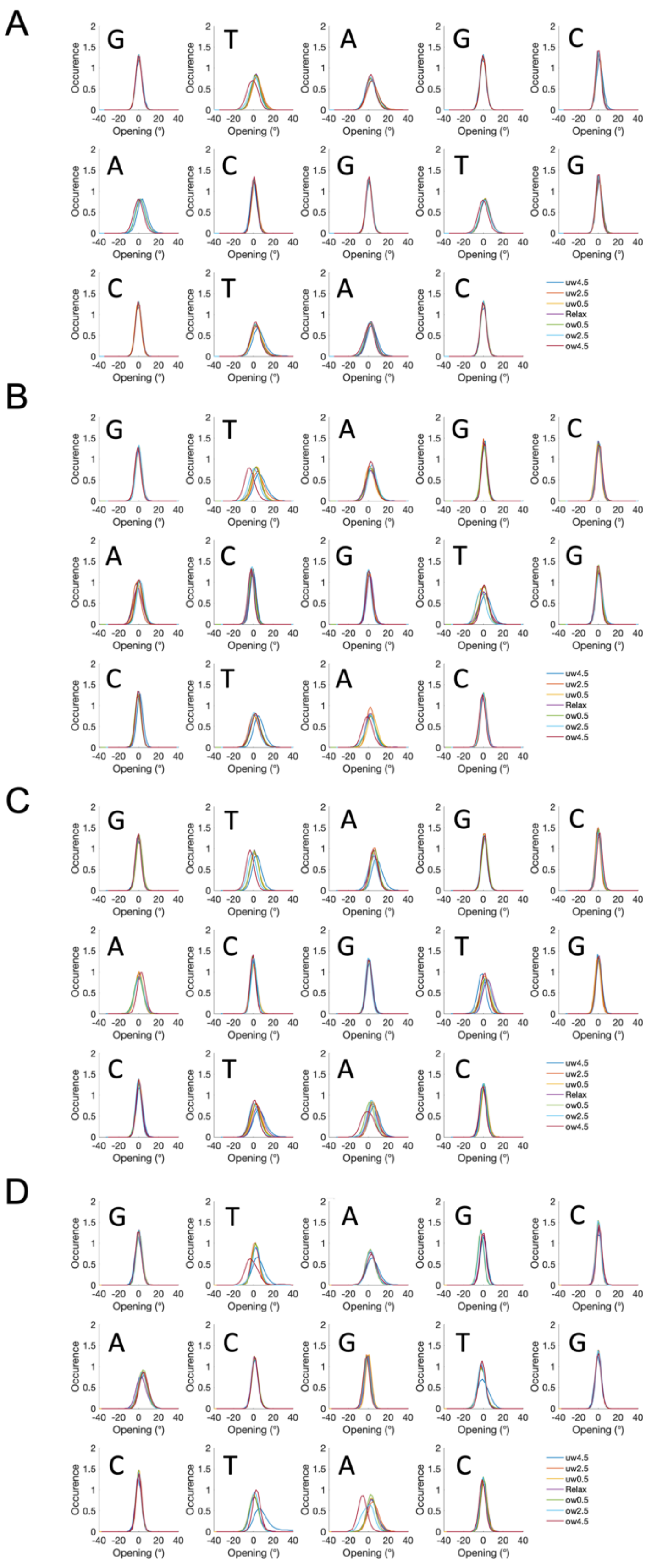

5'-GGCGA-**GTAGCACGTGCTAC**-TCGC-3'

Figure S4: Opening distributions for b.p. of the restrained region for different torsional regimes. **A.** DNA, **B.** MycMax-DNA, **C.** MadMax-DNA, **D.** MaxMax-DNA.

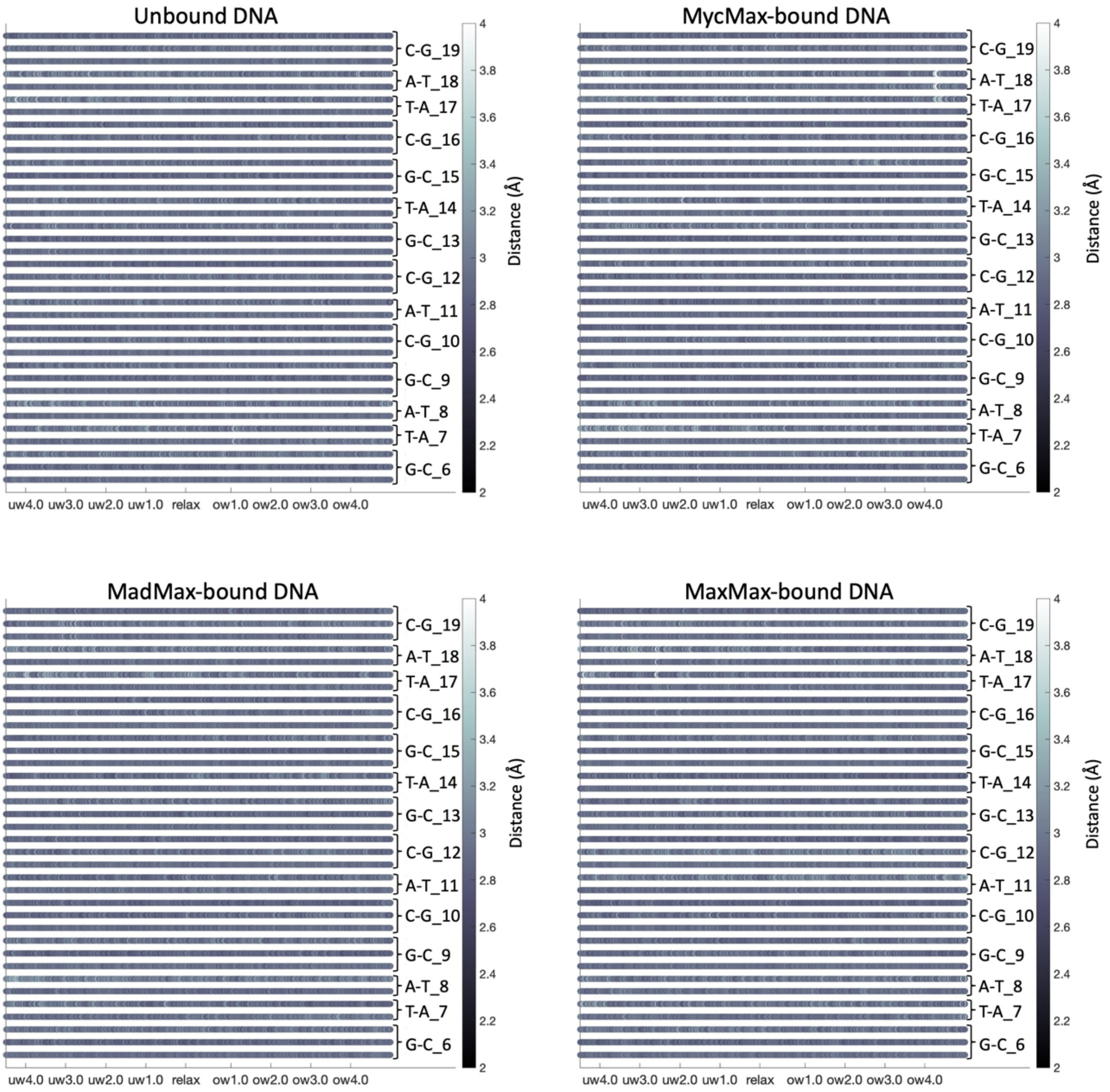

Figure S5: Evolution of hydrogen bond distances between heavy atoms of the b.ps within the restraint region (GTAGCACGTGCTAC) along the different torsional regimes for unbound DNA and MycMax-, MadMax and MaxMax-bound DNA. Distances are denoted with a colorbar.

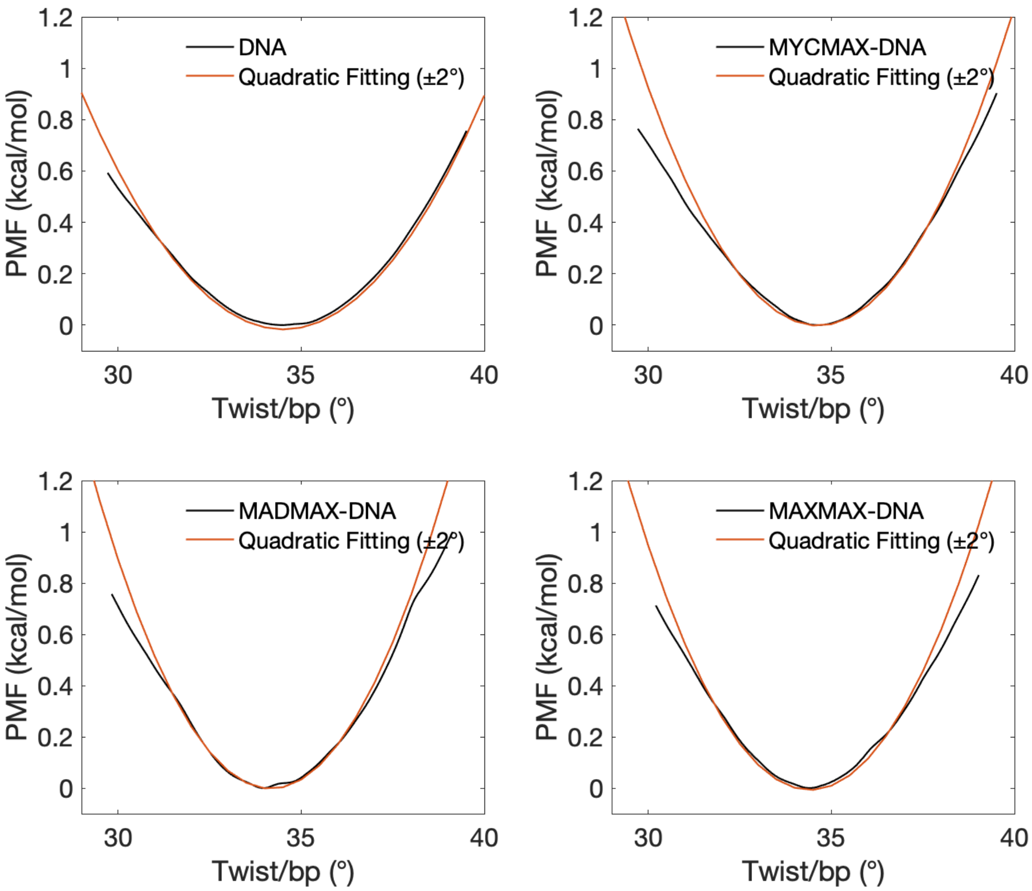

Figure S6: Quadratic regression (±2°) of PMF profiles of unbound DNA and MycMax-, MadMax- and MaxMax bound DNA is shown in orange lines.

Table S1: Calculated average relaxed twists, torsional constants 'K', torsional moduli 'C', and torsional persistence lengths 'P' for unbound DNA BHLH bound DNA.

	DNA	MycMax-DNA	MadMax-DNA	MaxMax-DNA
Relaxed twist (°)	34.5	34.7	34.0	34.4
K (kcal/mol*deg^2)	0.059	0.081	0.089	0.087
C (nm)	111	153	168	163

Supplementary figures S7-S12 are also available as separate supplementary figures if a reader would like to investigate them closer.

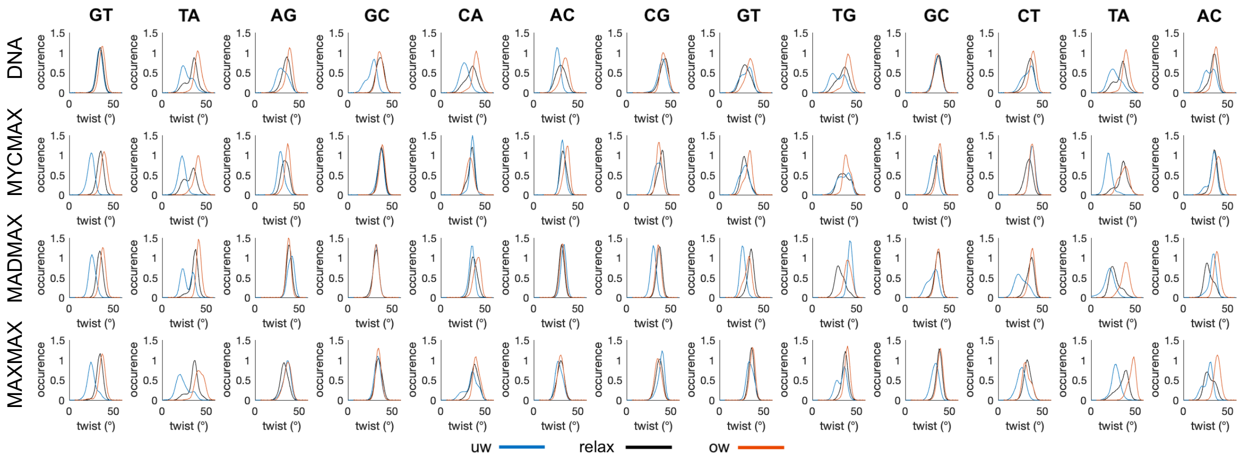

Figure S7: Twist distributions of the restrained DNA region (GTAGCACGTGCTAC) for underwound (-4.5), relaxed and overwound (+4.5) state.

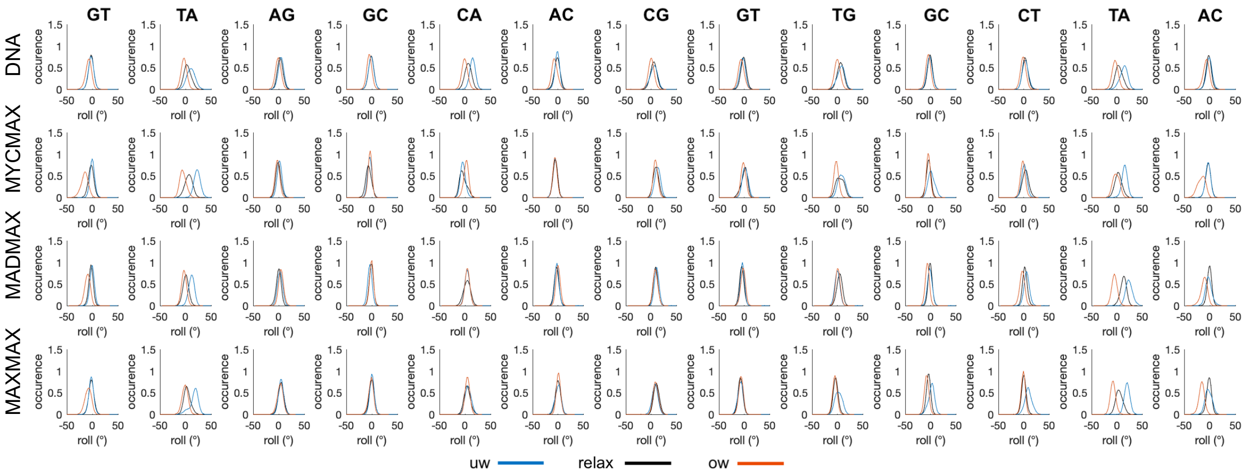

Figure S8: Roll distributions of the restrained DNA region (GTAGCACGTGCTAC) for underwound (-4.5), relaxed and overwound (+4.5) state.

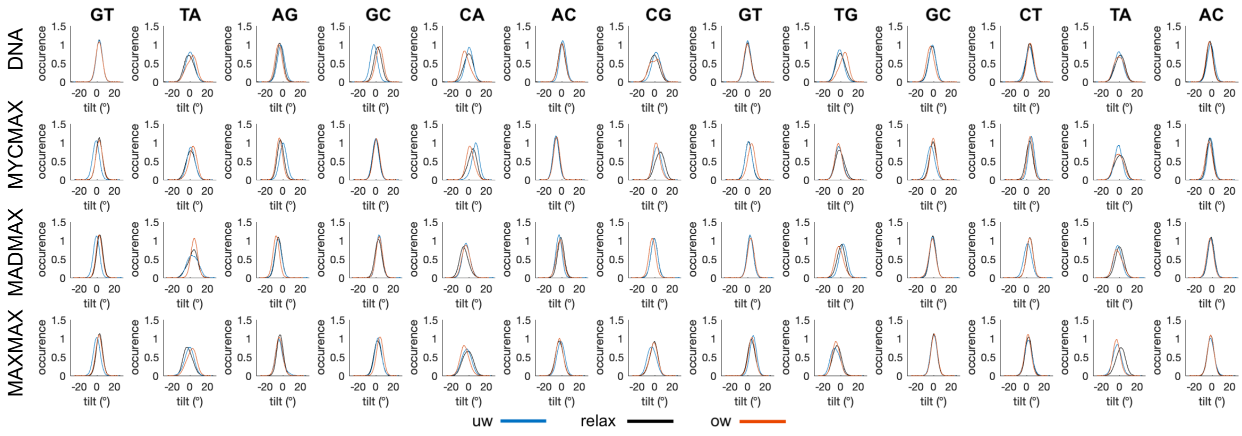

Figure S9: Tilt distributions of the restrained DNA region (GTAGCACGTGCTAC) for underwound (-4.5), relaxed and overwound (+4.5) state.

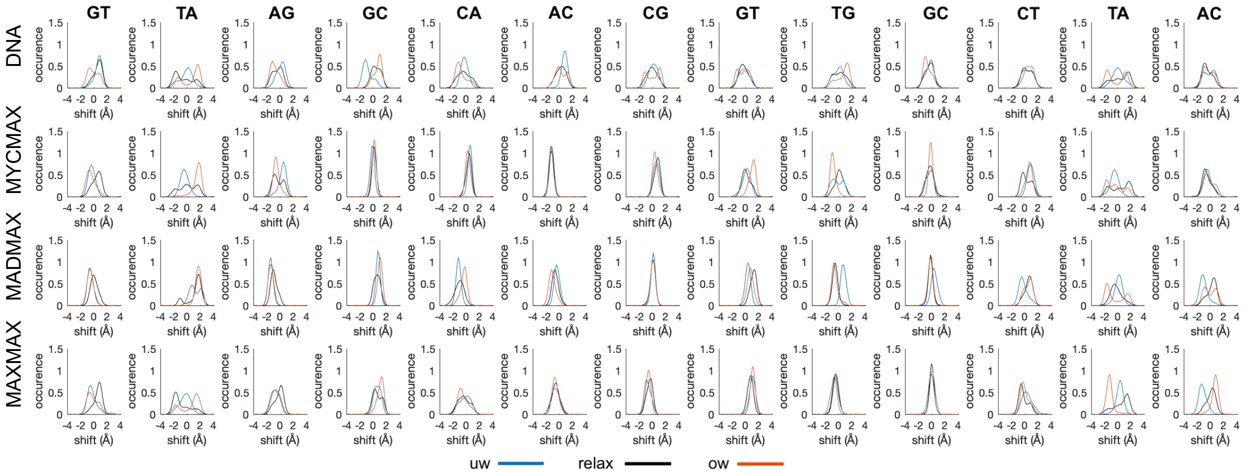

Figure S10: Shift distributions of the restrained DNA region (GTAGCACGTGCTAC) for underwound (-4.5), relaxed and overwound (+4.5) state.

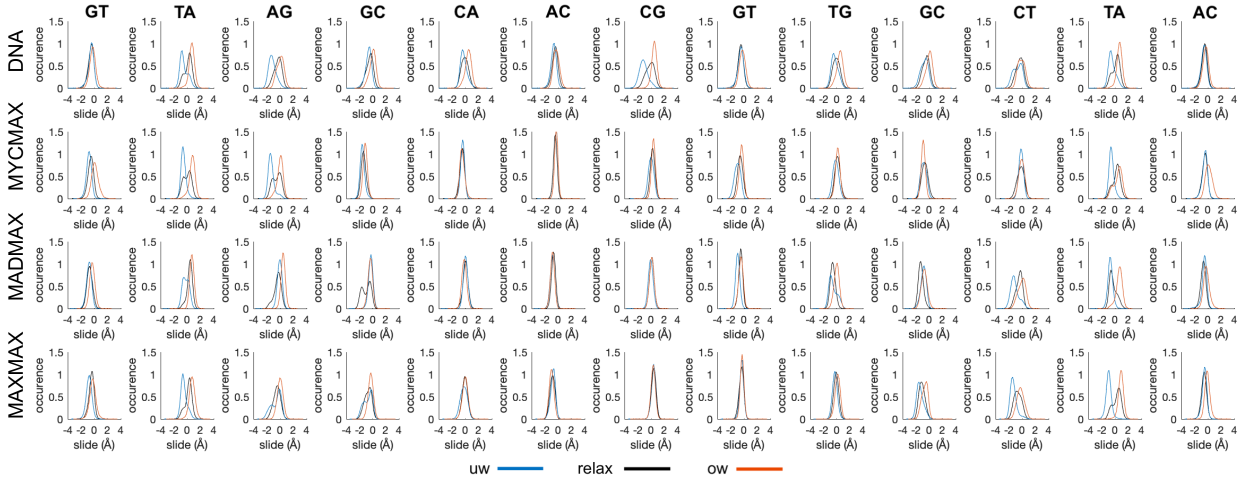

Figure S11: Slide distributions of the restrained DNA region (GTAGCACGTGCTAC) for underwound (-4.5), relaxed and overwound (+4.5) state.

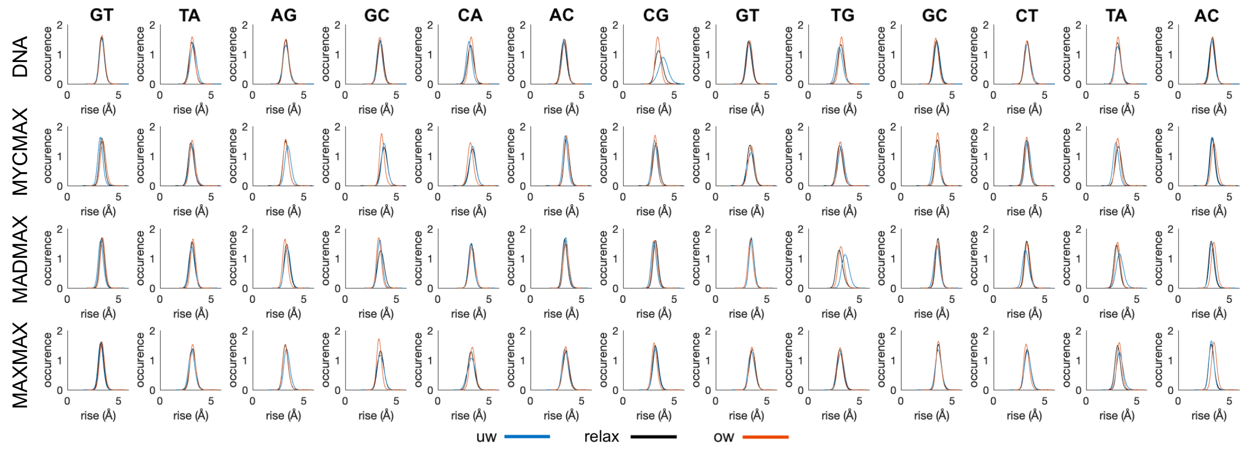

Figure S12: Rise distributions of the restrained DNA region (GTAGCACGTGCTAC) for underwound (-4.5), relaxed and overwound (+4.5) state.

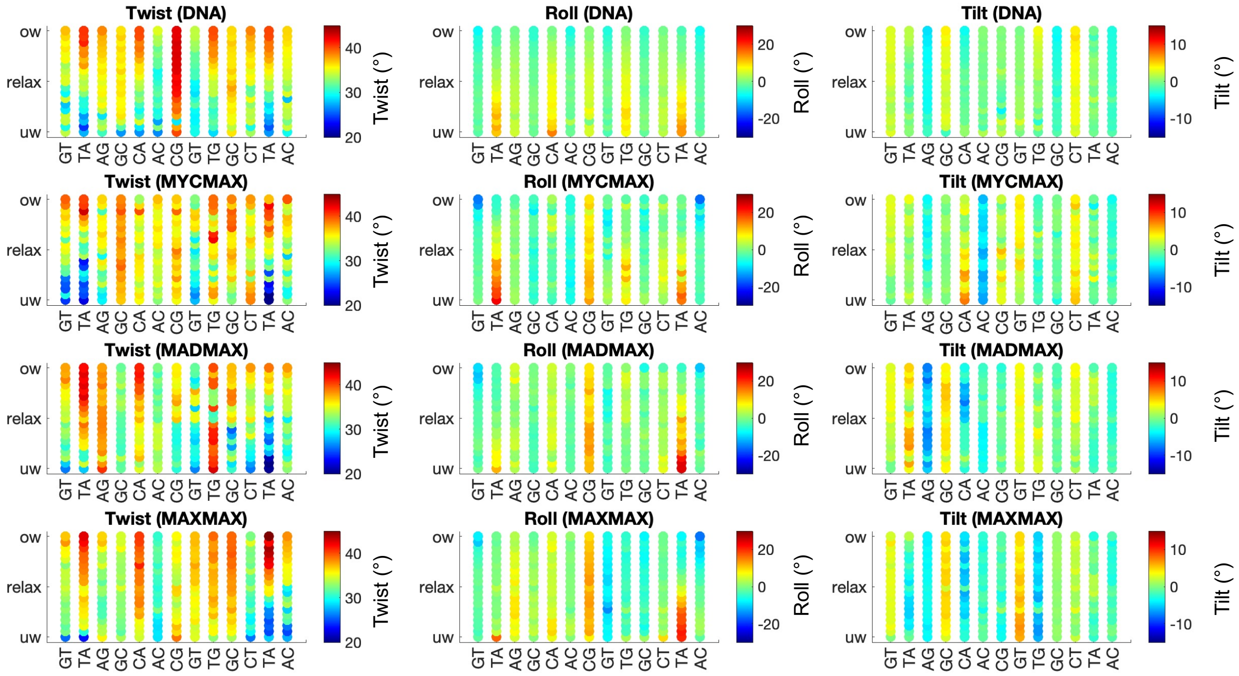

Figure S13: Change in average twist, roll and tilt of b.p. steps of the restrained DNA region (GTAGCACGTGCTAC) along the torsional regimes denoted with a colorbar.

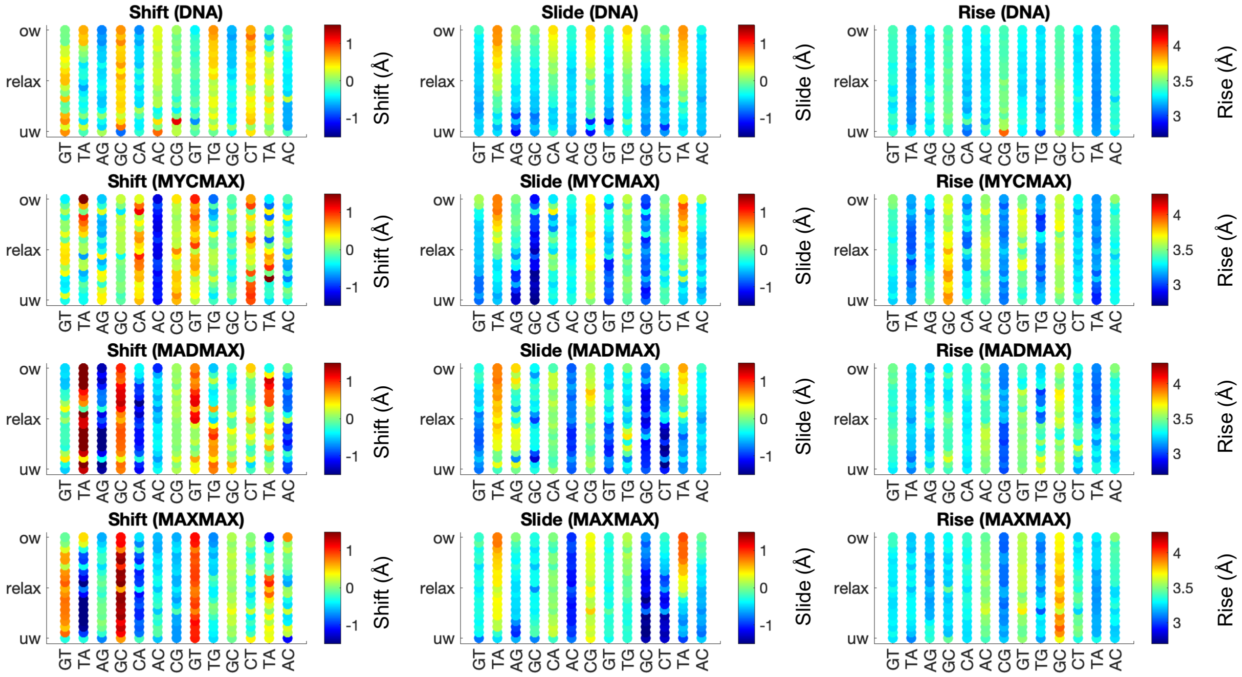

Figure S14: Change in average shift, slide and rise of b.p. steps of the restrained DNA region (GTAGCACGTGCTAC) along the torsional regimes denoted with a colorbar.

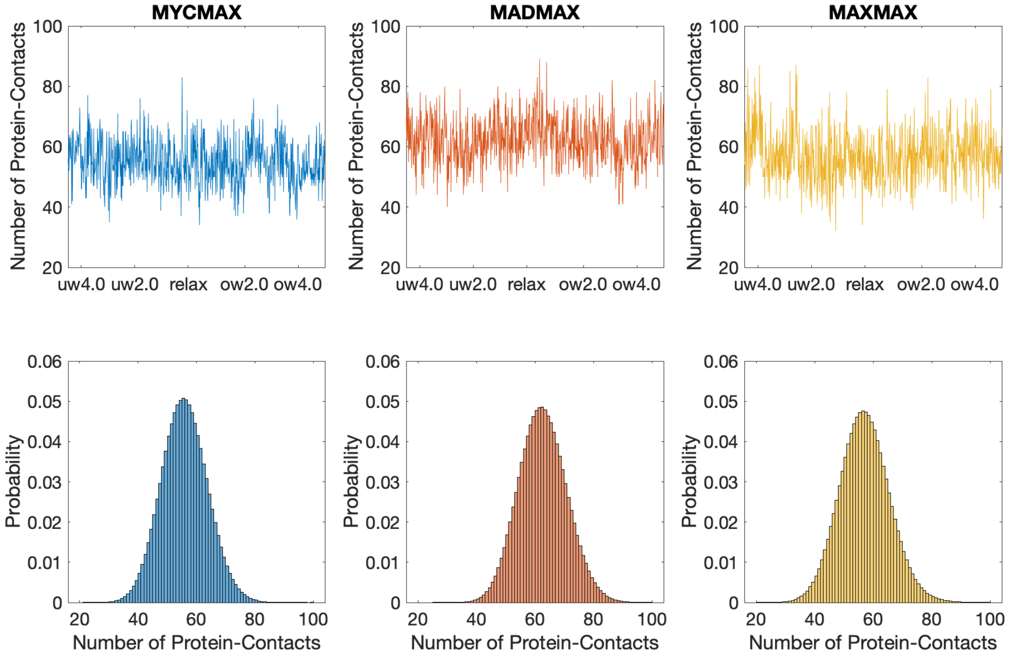

Figure S15: Upper panel: fluctuations of the total number of protein-DNA contacts along the torsional regimes. Bottom panel: distributions of the total number of protein-DNA contacts for the different BHLH-DNA complexes.

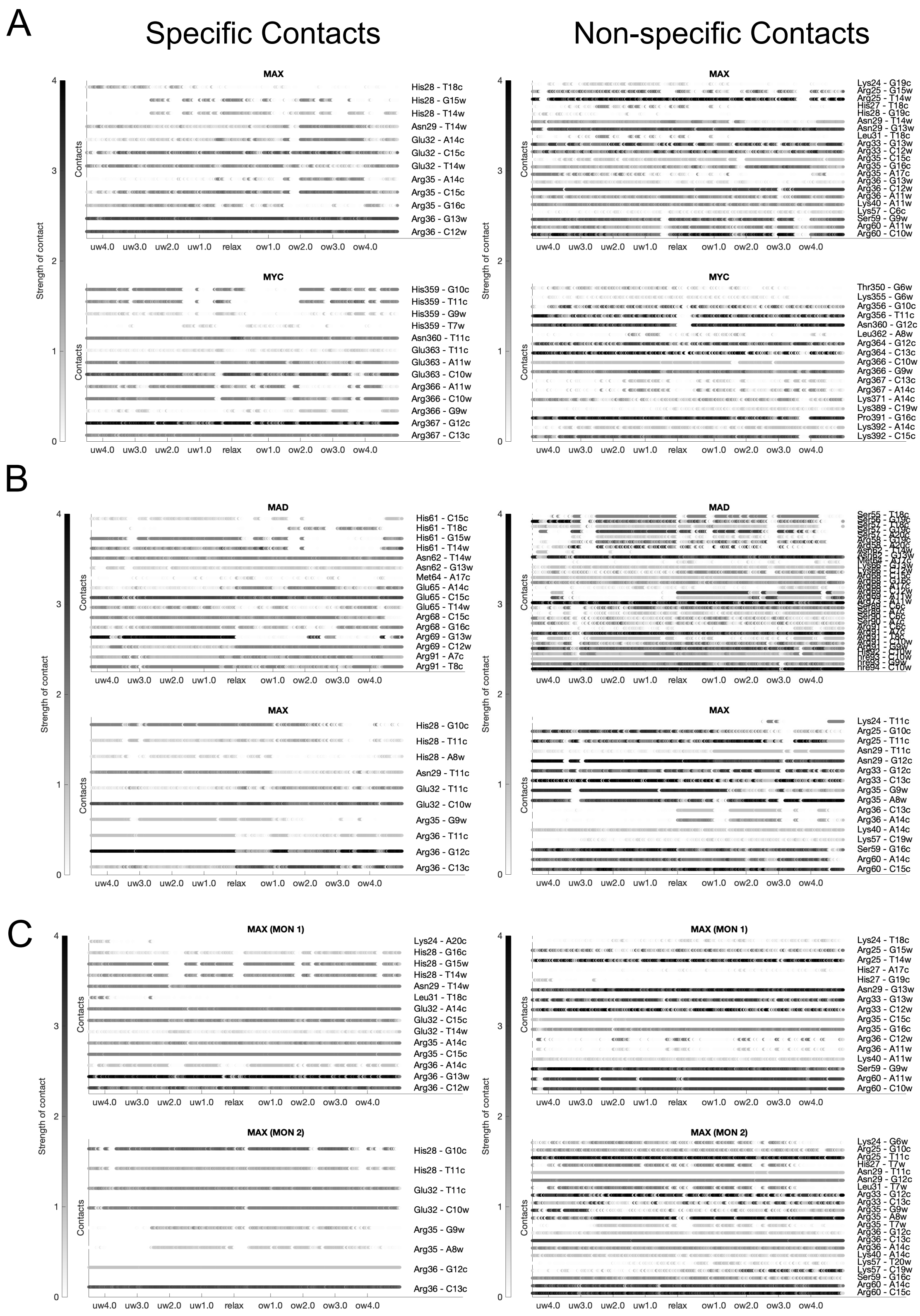

W-Strand 5’- G G C G A G T A G C A C G T G C T A C T C G C -3’

1 2 3 4 5 6 7 8 9 10 11 12 13 14 15 16 17 18 19 20 21 22 23

C-Strand 3’- C C G C T C A T C G T G C A C G A T G A G C G -5’

Figure S16: Dynamic interactions maps of specific and non-specific contacts along the different torsional regimes for **A.** MycMax, **B.** MadMax and **C.** MaxMax. The contacts between pairs of residues are characterized by strength and occurrence

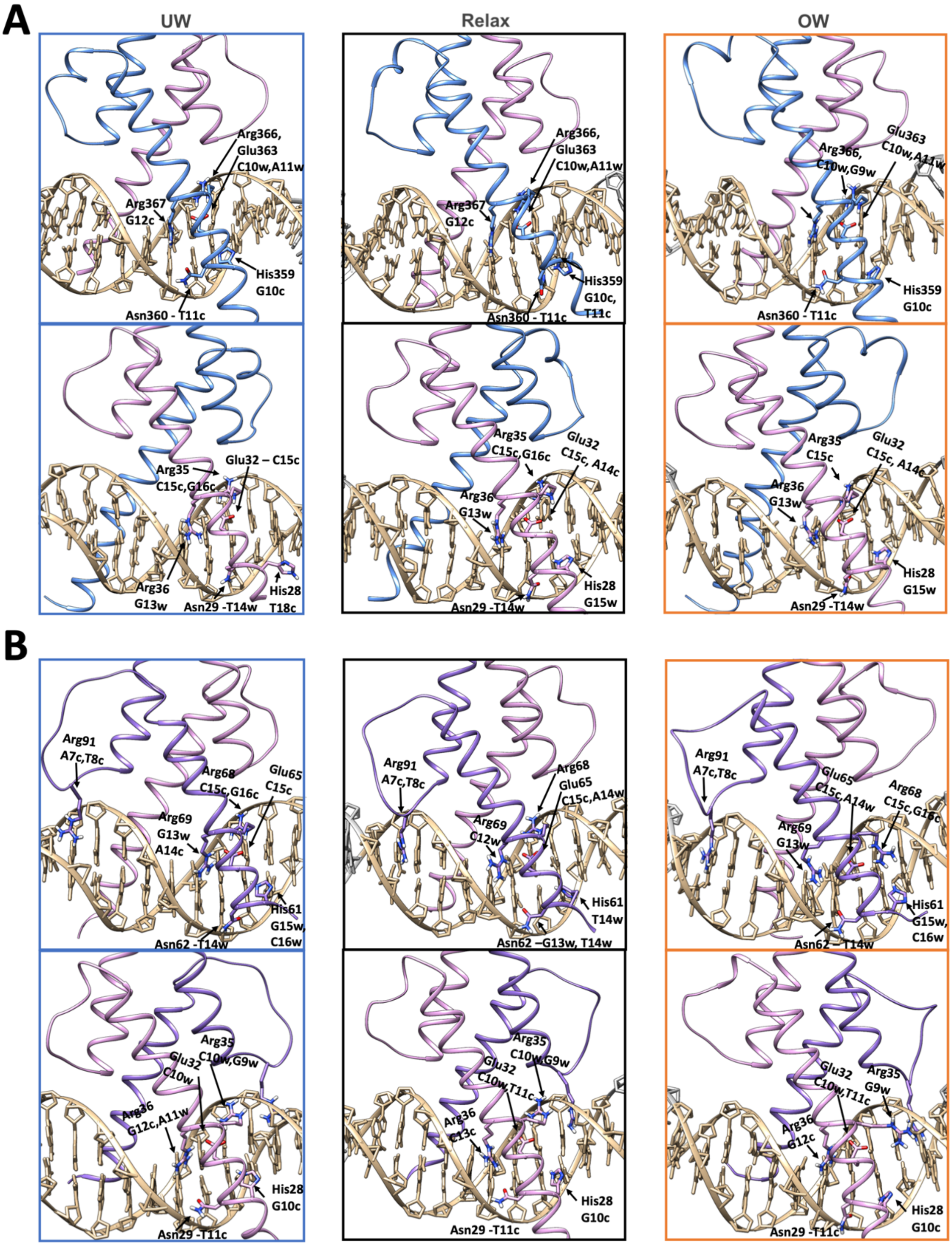
A
UW
Relax
OW
Arg366,
Glu363
C10w,A11w
Arg367
G12c
His359
G10c
Asn360 - T11c
Arg366,
Glu363
C10w,A11w
Arg367
G12c
His359
G10c,
T11c
Asn360 - T11c
Glu363
C10w,A11w
Arg366,
C10w,G9w
His359
G10c
Asn360 - T11c
Glu32 – C15c
Arg35
C15c,G16c
Arg36
G13w
Asn29 -T14w
His28
T18c
Arg35
C15c,G16c
Glu32
C15c, A14c
Arg36
G13w
His28
G15w
Asn29 -T14w
Arg35
C15c
Glu32
C15c, A14c
Arg36
G13w
His28
G15w
Asn29 -T14w
B
Arg91
A7c,T8c
Arg68
C15c,G16c
Glu65
C15c
Arg69
G13w
A14c
His61
G15w,
C16w
Asn62 -T14w
Arg91
A7c,T8c
Arg68
Glu65
C15c,A14w
Arg69
C12w
His61
T14w
Asn62 –G13w, T14w
Arg91
A7c,T8c
Glu65
C15c,A14w
Arg68
C15c,G16c
Arg69
G13w
His61
G15w,
C16w
Asn62 -T14w
Arg35
C10w,G9w
Glu32
C10w
Arg36
G12c,A11w
His28
G10c
Asn29 -T11c
Arg35
C10w,G9w
Glu32
C10w,T11c
Arg36
C13c
His28
G10c
Asn29 -T11c
Glu32
C10w,T11c
Arg35
G9w
Arg36
G12c
His28
G10c
Asn29 -T11c

C

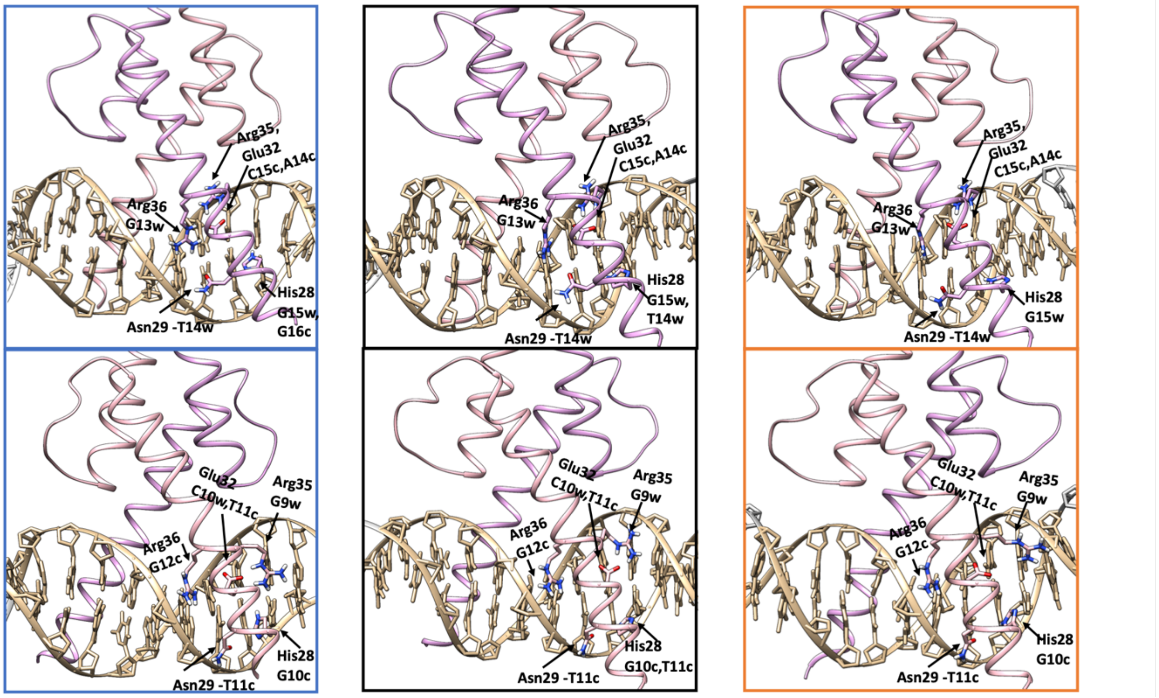

Figure S17: BHLH-DNA contacts exploited by the five-residues motif (**HN**xx**E**xx**RR**) for underwinding regime (-4.5°/bp), relaxed state and overwinding regime (+4.5°/bp). Restrained DNA region (GTAGCACGTGCTAC) is denoted with tan colour. **A.** Upper panel: Myc-DNA contacts. Bottom panel: Max-DNA contacts. **B.** Upper panel: Mad-DNA contacts. Specific contacts exploited by Mad loop is also shown. Bottom panel: Max-DNA contacts. **C.** Upper panel: Max(monomer 1)-DNA contacts. Bottom panel: Max(monomer 2)-DNA contacts.

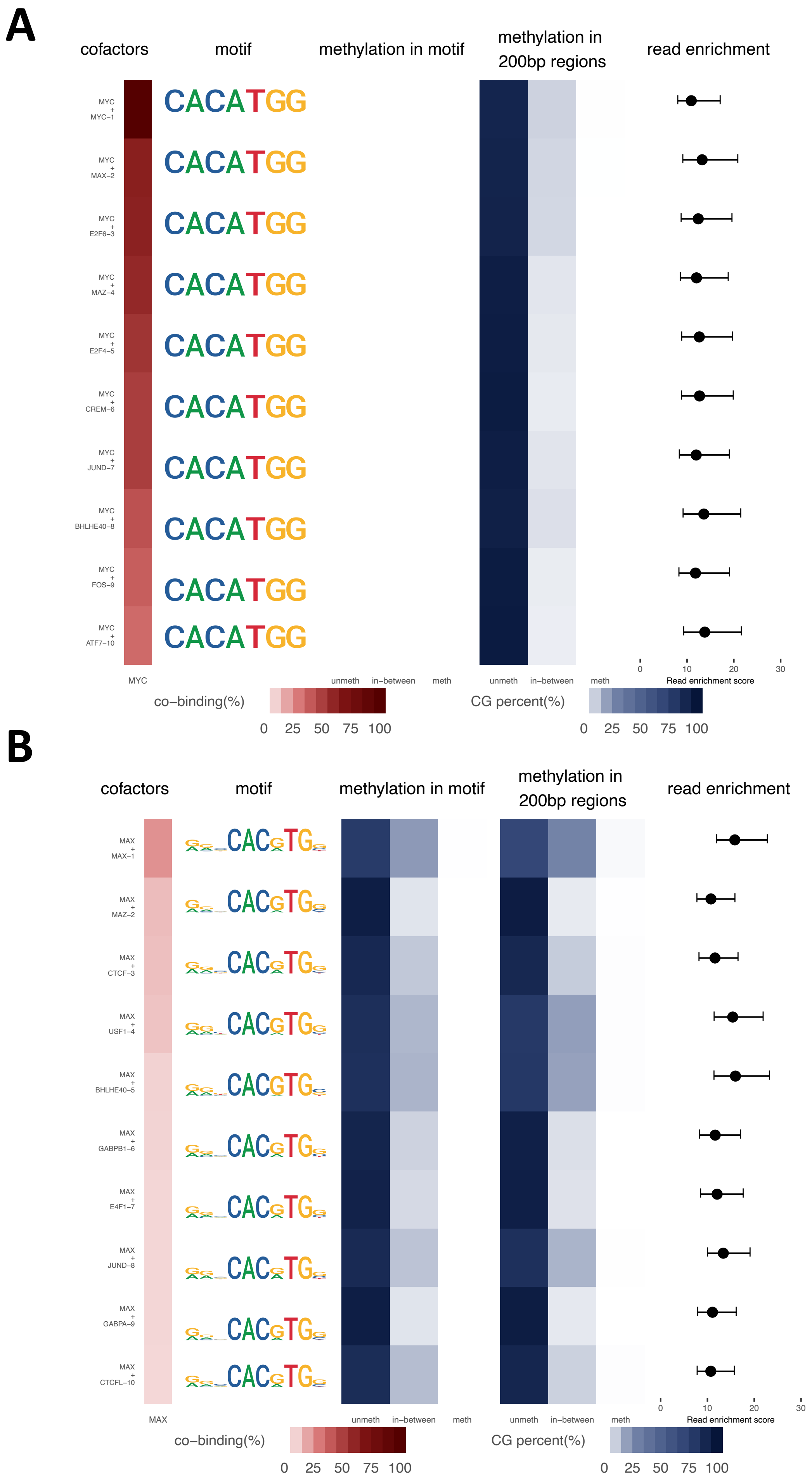
A
cofactors
motif
methylation in motif
methylation in 200bp regions
read enrichment
MYC + MYC−1
MYC + MAX−2
MYC + E2F6−3
MYC + MAZ−4
MYC + E2F4−5
MYC + CREM−6
MYC + JUND−7
MYC + BHLHE40−8
MYC + FOS−9
MYC + ATF7−10
CACATGG
MYC
unmeth
in−between
meth
0
10
20
30
Read enrichment score
co−binding(%)
CG percent(%)
0 25 50 75 100
B
cofactors
motif
methylation in motif
methylation in 200bp regions
read enrichment
MAX + MAX−1
MAX + MAZ−2
MAX + CTCF−3
MAX + USF1−4
MAX + BHLHE40−5
MAX + GABPB1−6
MAX + E4F1−7
MAX + JUND−8
MAX + GABPA−9
MAX + CTCFL−10
MAX
unmeth
in−between
meth
0
10
20
30
Read enrichment score
co−binding(%)
CG percent(%)
0 25 50 75 100

Figure S18: A. Myc and **B.** Max co-factors summary as reported by TFregulomeR. Information reported are, from left to right, the name of transcription factors detected as potential co-factors to either Myc or Max, with the corresponding co-binding percentage in shade of red; the sequence motif for the main protein of interest (Myc or Max); the methylation percentage of cytosine inside the binding motif and in a window of 200bp around the binding motif, represented in shade of blue; the reads enrichment from the ChIP-seq profile of the considered proteins.
