# Supplementary figures and images for: Homologous basic helix–loop–helix transcription factors induce distinct deformations of torsionally-stressed DNA: a potential transcription regulation mechanism
Source: QRB Discov. 2022 Jun 10;3:e4. doi: 10.1017/qrd.2022.5 (PMC10392670; doi:10.1017/qrd.2022.5)

DNA

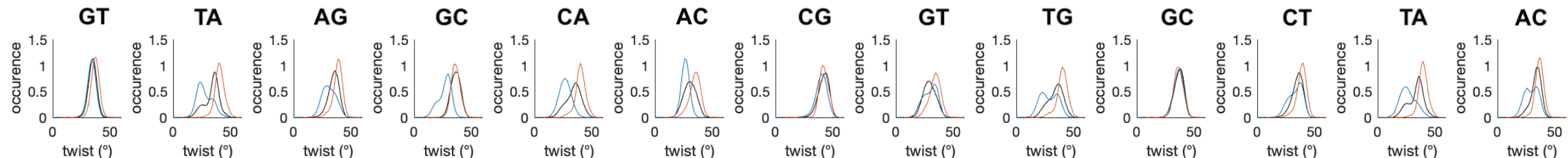

MYCMAx

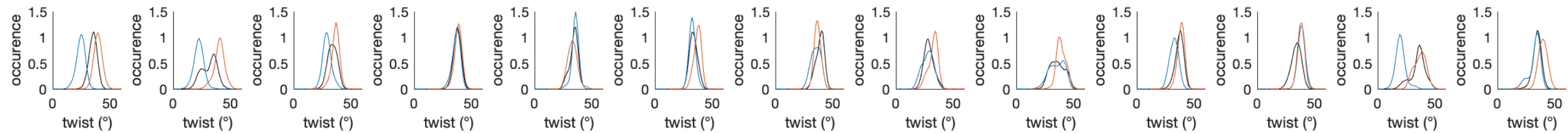

MADMAx

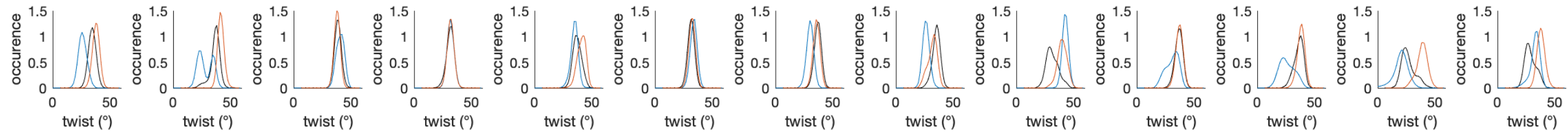

MAXMAx

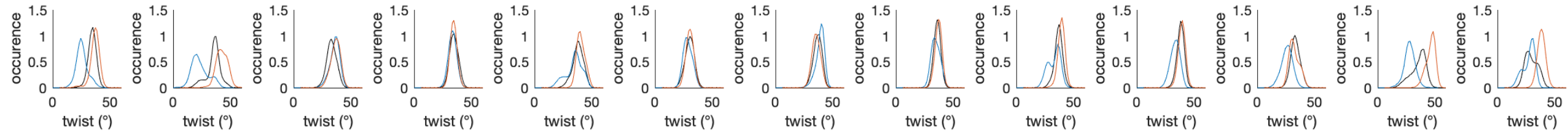

uw — relax — ow

Supplement: Supplementary file 1 [file S2633289222000059sup001.zip › S2633289222000059sup002.pdf]

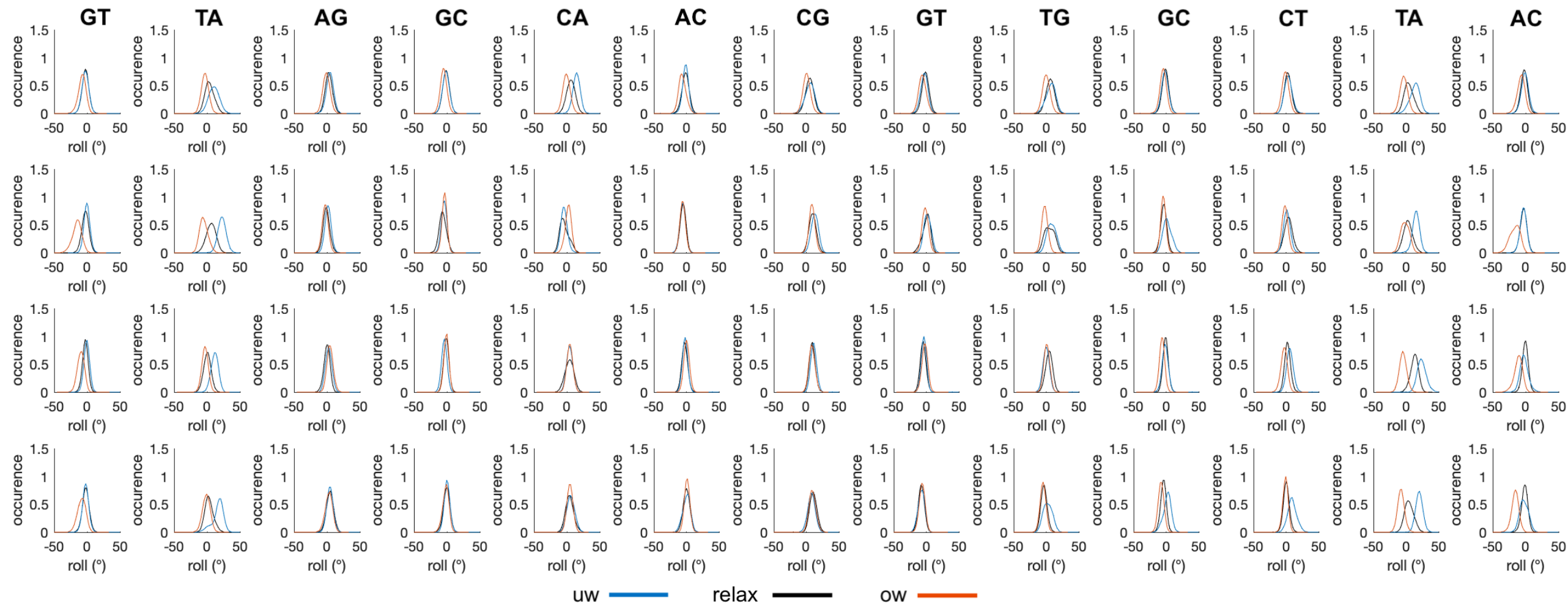

Supplement: Supplementary file 1 [file S2633289222000059sup001.zip › S2633289222000059sup003.pdf]

DNA

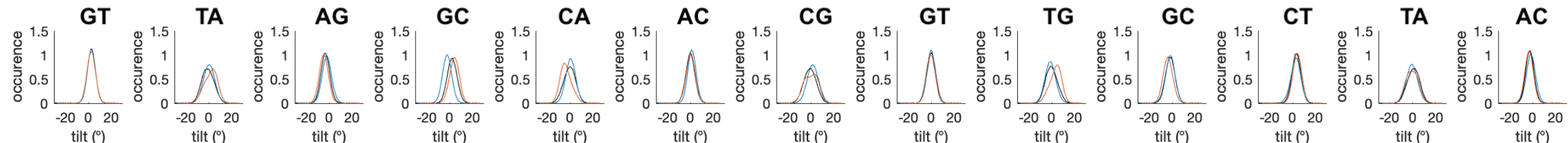

MYCMAx

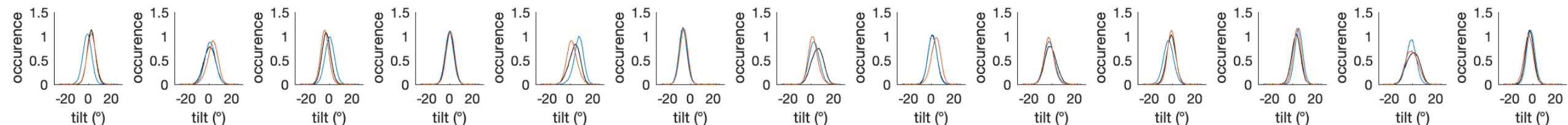

MADMAx

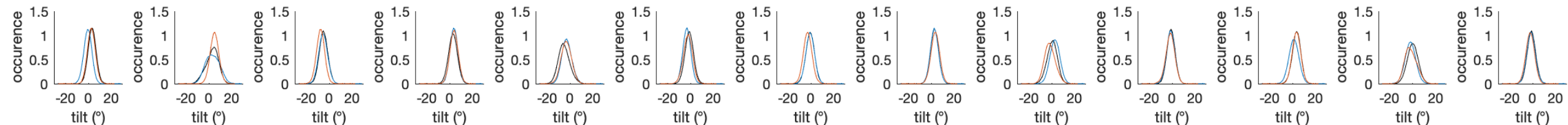

MAXMAx

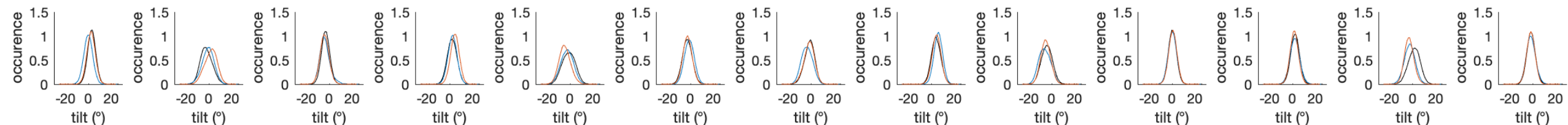

uw — relax — ow

Supplement: Supplementary file 1 [file S2633289222000059sup001.zip › S2633289222000059sup004.pdf]

DNA

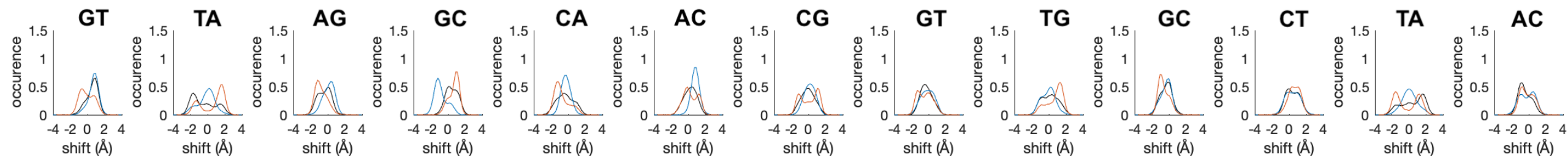

MYCMAx

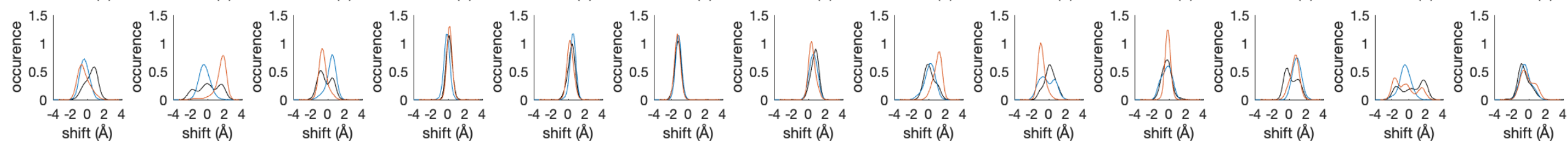

MADMAx

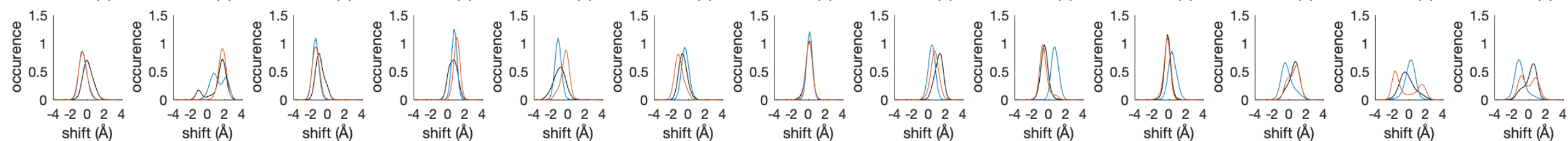

MAXMAx

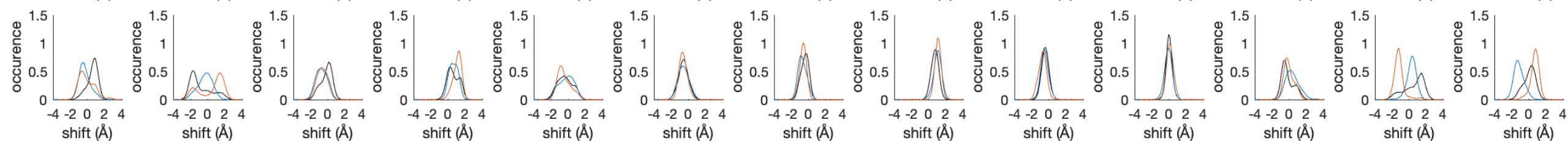

uw — relax — ow

Supplement: Supplementary file 1 [file S2633289222000059sup001.zip › S2633289222000059sup005.pdf]

DNA

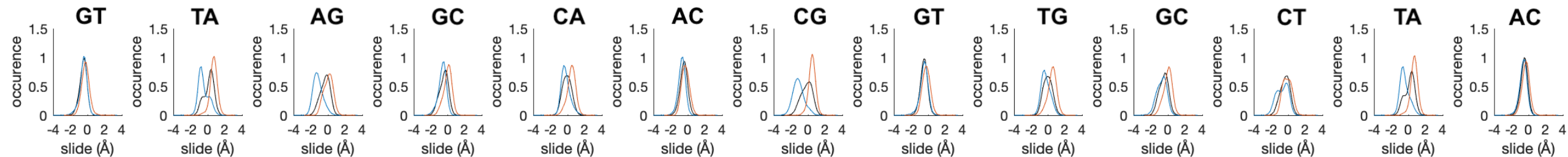

MYCMAx

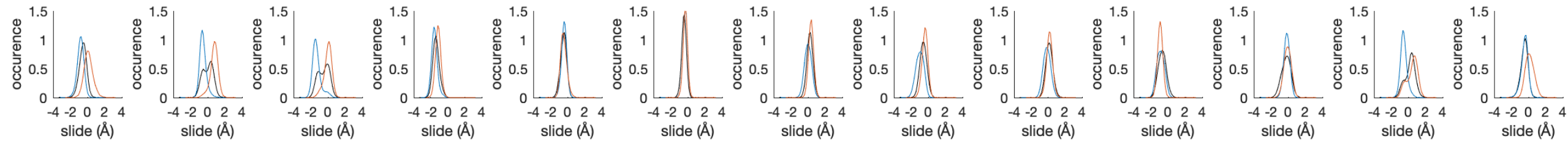

MADMAx

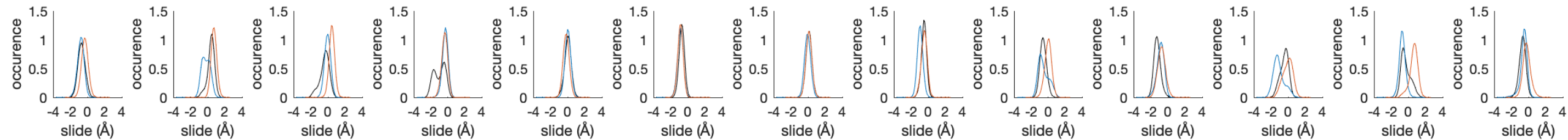

MAXMAx

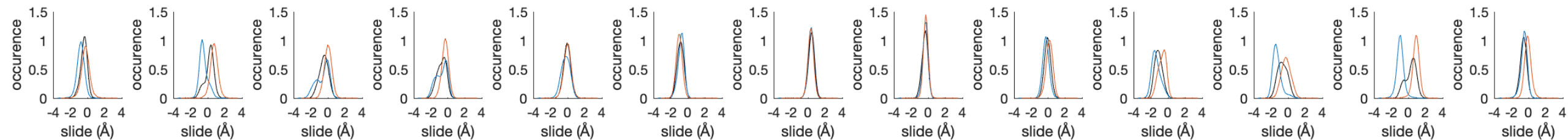

uw — relax — ow

Supplement: Supplementary file 1 [file S2633289222000059sup001.zip › S2633289222000059sup006.pdf]

MAXMAX  
MADMAX  
MYCMAX  
DNA

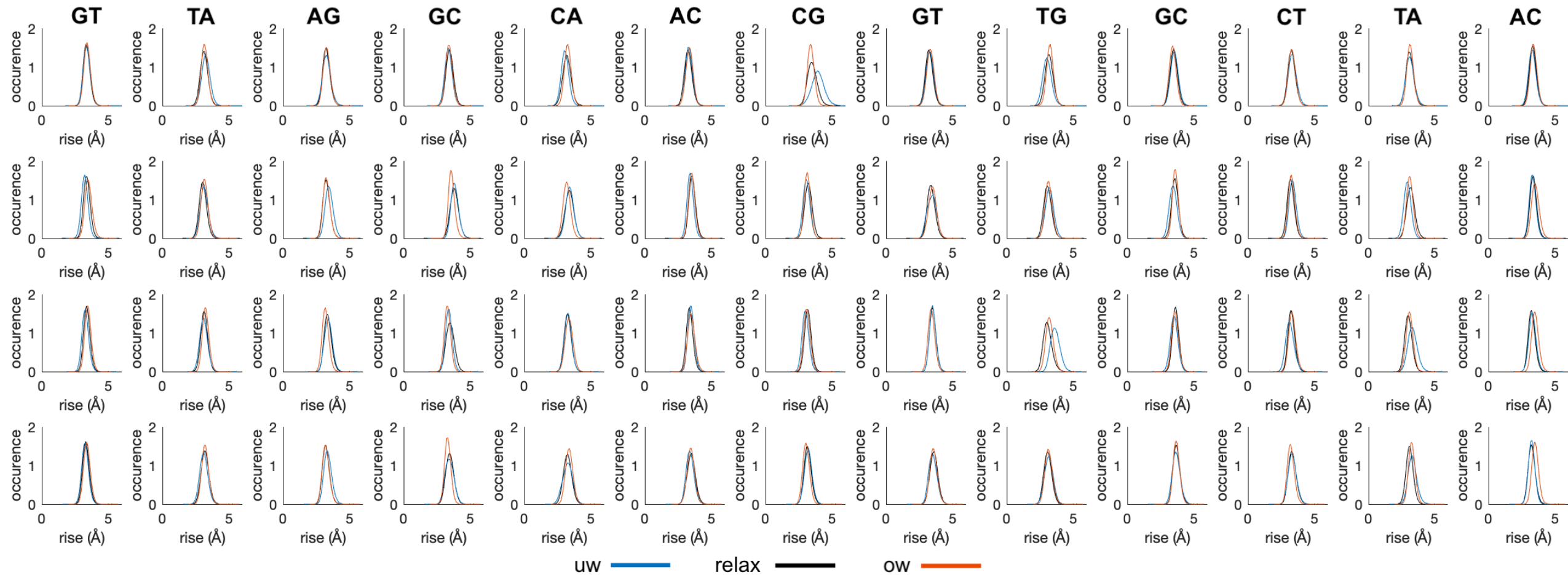

Supplement: Supplementary file 1 [file S2633289222000059sup001.zip › S2633289222000059sup007.pdf]
